# Peer reviewers' willingness to review, their recommendations and quality of reviews after the Finnish Medical Journal switched from single-blind to double-blind peer review

**DOI:** 10.1186/s41073-023-00140-6

**Published:** 2023-10-24

**Authors:** Piitu Parmanne, Joonas Laajava, Noora Järvinen, Terttu Harju, Mauri Marttunen, Pertti Saloheimo

**Affiliations:** 1 Finnish Medical Association, Helsinki, Finland; 2 University of Helsinki, Helsinki, Finland; 3 Finnish Medical Journal, Helsinki, Finland; 4 Respiratory Medicine Research Unit, Department of Medicine, University of Oulu, Oulu, Finland; 5 Medical Research Center, Oulu University Hospital, Oulu, Finland; 6 OYS Somatics, Internal Medicine Centre, Pulmonary Outpatient Clinic, Oulu University Hospital, Oulu, Finland; 7 Department of Adolescent Psychiatry, Helsinki University Hospital and University of Helsinki, Helsinki, Finland; 8 Department of Public Health Solutions, Finnish Institute for Health and Welfare in Finland (THL), Helsinki, Finland

**Keywords:** Peer review, Quality of peer review, Single-blind peer review, Double-blind peer review, Scientific publication

## Abstract

**Background:**

There is a power imbalance between authors and reviewers in single-blind peer review. We explored how switching from single-blind to double-blind peer review affected 1) the willingness of experts to review, 2) their publication recommendations, and 3) the quality of review reports.

**Methods:**

The Finnish Medical Journal switched from single-blind to double-blind peer review in September 2017. The proportion of review invitations that resulted in a received review report was counted. The reviewers’ recommendations of “accept as is”, “minor revision”, “major revision” or “reject” were explored. The content of the reviews was assessed by two experienced reviewers using the Review Quality Instrument modified to apply to both original research and review manuscripts. The study material comprised reviews submitted from September 2017 to February 2018. The controls were the reviews submitted between September 2015 and February 2016 and between September 2016 and February 2017. The reviewers’ recommendations and the scorings of quality assessments were tested with the Chi square test, and the means of quality assessments with the independent-samples t test.

**Results:**

A total of 118 double-blind first-round reviews of 59 manuscripts were compared with 232 single-blind first-round reviews of 116 manuscripts. The proportion of successful review invitations when reviewing single-blinded was 67%, and when reviewing double-blinded, 66%. When reviewing double-blinded, the reviewers recommended accept as is or minor revision less often than during the control period (59% vs. 73%), and major revision or rejection more often (41% vs 27%, *P* = 0.010). For the quality assessment, 116 reviews from the double-blind period were compared with 104 reviews conducted between September 2016 and February 2017. On a 1–5 scale (1 poor, 5 excellent), double-blind reviews received higher overall proportion of ratings of 4 and 5 than single-blind reviews (56% vs. 49%, *P* < 0.001). Means for the overall quality of double-blind reviews were 3.38 (IQR, 3.33–3.44) vs. 3.22 (3.17–3.28; *P* < 0.001) for single-blind reviews.

**Conclusions:**

The quality of the reviews conducted double-blind was better than of those conducted single-blind. Switching to double-blind review did not alter the reviewers’ willingness to review. The reviewers became slightly more critical.

## Background

Peer review is considered a key element of a scientific journal, but many flaws have been identified. It is slow, poor in detecting fraud, highly subjective, prone to bias, expensive, and easily abused [1, 2]. The traditional model is single-blind peer review: the reviewers know the identity of the authors but the authors do not know who the reviewers are. This model is common in biomedical and natural sciences [3, 4].

In addition to the weaknesses described above, there is a serious power imbalance in single-blind peer review. There is plenty of evidence that author characteristics, such as prestige, affiliation, nationality, language, and gender may affect the reviewers’ opinions and assessments of the manuscripts [3, 5]. The study by Huber et al. clearly shows the bias associated with the author characteristics. They invited more than 3,300 potential reviewers to review the same manuscript, either showing a very prominent corresponding author, or a relatively unknown researcher as corresponding author, or anonymized. When the prominent researcher was shown as the corresponding author, 23% of the reviewers recommended rejection, while for anonymized manuscript 48% did so, and when the less known author was shown, rejection was recommended by 65% of the reviewers [6].

Innovations aiming to tackle at least some of these biases include two opposite formats of peer review: double-blind peer review and open peer review. In double-blind peer review neither authors nor reviewers know the identity of the others. Double-blinding should reduce the status bias and other biases of single-blind peer review [1, 3–6]. The model was first introduced in the social sciences [4] and is more common in many other fields of science than in biomedicine, although it is gaining ground in biomedical journals, too. In open peer review the names of the reviewers are revealed to the authors and the names of the authors to the reviewers. Some journals also publish the reviewers’ names and comments as well as the previous versions of the manuscripts [4, 7].

The Finnish Medical Journal is one of the two major general medical journals in Finland. The journal is published by the Finnish Medical Association, and the members receive the journal as member benefit. Other health care professionals and institutions can subscribe for the journal. The journal is published in Finnish, with English summaries online. As a journal published in a national language, it is not indexed internationally and, thus, has no impact factor. The journal annually publishes c. 120 peer-reviewed articles, including original research articles, review articles and case reports. In addition to those, editorials, opinion pieces and letters to the editor, as well as news and feature articles are published. There are no article processing charges.

Most review articles are commissioned and both non-commissioned and commissioned reviews, as well as original research articles and case-reports, are externally peer reviewed by at least two reviewers. The reviewers are medical or other professionals with scientific training, and they are selected by the journal’s associate editors. Due to the language, the pool of potential reviewers is limited to Finnish speaking experts. There are c. 30,000 licensed physicians in Finland, about 25% of them with a doctorate (data from the Finnish Medical Association).

In September 2017, the Finnish Medical Journal switched from single-blind to double-blind peer review. After the switch to double-blind peer review, a separate title page including the names and affiliations of the authors was required, and the main text without this information was instructed to be submitted as a separate file. The authors were instructed to avoid such expressions as “our previous study” and “our hospital”. Any acknowledgements that might have revealed, e.g., the institutions of the authors were removed before peer review by the editorial staff.

We explored how switching to double-blind peer review affected 1) the willingness of experts to review, 2) their publication recommendations, and 3) the quality of review reports.

## Methods

**Fig. 1:**
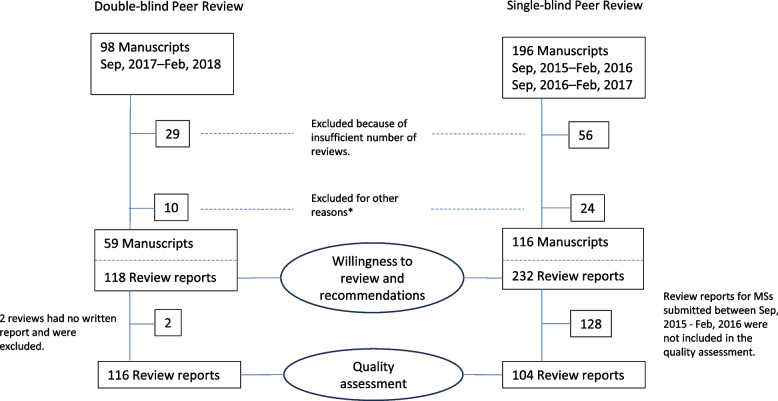
Data flow chart of double-blind and single-blind peer reviews and analyses of the reviewers’ willingness to review, their publication recommendations and quality assessment of review reports. *Review was incomplete, one of the reviewers was an associate editor for the journal, or the reviewer clearly knew the author during the double-blind period

Our material comprised reviews submitted to the Finnish Medical Journal between September 1^st^, 2017, and February 28^th^, 2018. The controls were the reviews submitted between September 2015 and February 2016 and between September 2016 and February 2017, i.e., the corresponding months of the respective years. The reviews on all manuscripts with at least 2 first-round reviews were included. In cases where there were more than 2 reviews for the manuscript, the first 2 reviews received were included (Fig. 1).

To explore the willingness of the reviewers to review, we calculated the proportion of review invitations that led to a received review, and how many invitations were needed to receive 2 reviews. We also explored how often the reviewers recommended accept as is, minor revision, major revision or reject.

In the quality analysis, review reports from the double-blind period were compared with those submitted between September 2016 and February 2017. The contents of the reports were independently assessed by two experienced reviewers (TH and MM) using the Review Quality Instrument (RQI) [8]. Thus, we obtained two scorings for each review report assessed. These reviewers had not conducted any of the reviews on the manuscripts in the study material. They were unaware of the peer review model used and the decisions made on the manuscripts. RQI was modified to apply to both original research manuscripts and review manuscripts. We modified “Did the reviewer discuss the importance of the research question?” to “Did the reviewer discuss the importance of the research question/topic of the review?”, “Did the reviewer identify the strengths and weaknesses of the method?” to “Did the reviewer identify the strengths and weaknesses of the method/literature search?”, and “Did the reviewer comment on the author’s interpretation of the results?” to “Did the reviewer comment on the author’s interpretation of the results/literature?”.

### Statistical methods

The reviewers’ recommendations were tested with the Chi square test. We assessed inter-rater reliability of two reviewers’ recommendations for the same manuscript with Fleiss’ Kappa [9], which is a measure for multiple independent raters, and values > 0 indicate agreement better than by chance.

For the review report scorings by the two reviewers (TH and MM), we assessed consistency of their scores with intraclass correlation coefficient (ICC) using a two-way mixed-effects model. ICC reflects a degree of correlation and agreement between two raters’ measurements of same group of subjects, and values > 0.75 indicate good reliability [10].

We classified the review report scorings to high (4 and 5) and others. The proportions on high scorings were tested with the Chi square test. The means of quality assessments were tested with the independent-samples t test. *P* < 0.05 was considered statistically significant. Data analysis was performed using IBM SPSS Statistics v29 (RRID:SCR_016479).

## Results

Reviews for 294 manuscripts had been submitted to the journal’s web server during the study and control periods. For 85 manuscripts, only 1 review had been submitted, and these were excluded. In addition, 34 manuscripts were excluded for various reasons: review was incomplete, one of the reviewers was an associate editor for the journal, or the reviewer clearly knew the author during the double-blind period. In our analysis, 118 reviews of 59 original research manuscripts and review manuscripts conducted double-blind by 114 individual reviewers were included. These were compared with 232 reviews of 116 manuscripts conducted single-blind by 213 reviewers, i.e., the reviewers knew the identity of the authors (Fig. 1). Fourteen reviewers reviewed during both the study and control periods. Assumed on the basis of their first names, 146 (47%) reviewers were female and 167 (53%) were male.

During the double-blind period, 180 review invitations led to 118 review reports, and during the control periods, 345 invitations to 232 review reports. Success rates for invitations were 66% and 67%, respectively. The median number of review invitations to obtain 2 reviews was 3 (IQR 2–4) during the double-blind period, and 3 (IQR 2–4) during the control periods. The range was 2 to 7 in both groups.

The level of agreement between two reviewers’ recommendations for the same manuscripts was positive indicating slight agreement (Fleiss’ kappa, 0.12; 95%CI, 0.02–0.23; *P* = 0.02). When performing a double-blinded review, the reviewers’ recommendations were slightly more negative than those of single-blinded reviewers’, but the difference in the distribution of recommendations was not statistically significant (*P* = 0.070, test for trend) (Table 1). When analyzed separatedly, major revision was recommended more often during the double-blind period than during the control period (33% vs. 23%, *P* = 0.040). Combined, accept as is or minor revision were recommended for 70 (59%) manuscripts during the double-blind period and for 169 (73%) during the single-blind period (*P* = 0.010). 54 (92%) manuscripts from the double-blind period and 106 (91%) from the single-blind period led to a published article.

**Table 1:** Reviewers’ recommendations when reviewing single-blind vs double-blind

Recommendation	Single-blind, n (%)	Double-blind, n (%)	*P* value^*^	Reviews assessed using RQI; single-blind, n (%)	Reviews assessed using RQI; double-blind, n (%)	*P* value^**^
**Accept as is**	28 (12)	10 (8)	0.307	12 (12)	9 (8)	0.341
**Minor revision**	141 (61)	60 (51)	0.076	60 (58)	59 (51)	0.310
**Major revision**	53 (23)	39 (33)	0.040	27 (26)	41 (35)	0.133
**Reject**	10 (4)	9 (8)	0.195	5 (5)	7 (6)	0.689
**Total**	232 (100)	118 (100)		104 (100.0)	116 (100.0)	

^*^P for trend, 0.070

^**^P for trend, 0.391

For the quality assessment 116 reviews were included (in 2 cases there was no written review) and compared to 104 reviews conducted between September 2016 and February 2017. We calculated the inter-rater reliability ICC of RQI scorings between the two independent reviewers (TH and MM). The ICC was moderate for all RQI items (ICC, 0.65; 95%CI, 0.61–0.68; *P* < 0.001) ranging from highest in item 4 (ICC, 0.76; 95%CI, 0.69–0.82; *P* < 0.001) to lowest in item 8 (ICC, 0.36; 95%CI, 0.16–0.51, *P* < 0.001).

The proportions of high quality scorings for RQI item 2 and overall were significantly higher when reviewing double-blinded. The reviewers more often discussed the originality of the manuscript (item 2) when reviewing double-blinded than when reviewing single-blinded: the proportions of scorings 4 and 5 on a 1–5 scale (1 poor, 5 excellent) were 39% and 26%, respectively (*P* = 0.003) (Table 2), and means were 2.90 (IQR, 2.72–3.07) vs 2.51 (2.33–2.69; *P* = 0.003) (Table 3). The other single questions did not show statistically significant differences, but the overall quality of the reviews conducted double-blind was significantly better than that of those conducted single-blind: overall proportions of all scores 4 and 5 were 55% and 49% (*P* < 0.001). The means were 3.38 (IQR, 3.33–3.44) vs 3.22 (3.17–3.28; *P* < 0.001).

**Table 2:** Quality assessment of the reviews conducted single-blind vs. double-blind; the proportions of quality ratings 4 and 5 and 95%CI 95 s

	**Single-blind** (n 104)	**Double-blind** (n 116)	***P* value**
1. Did the reviewer discuss the importance of the research question/topic of the review?	53% 46–60	62% 56–68	0.07
2. Did the reviewer discuss the originality of the MS?	26% 20–32	39% 33–45	0.003
3. Did the reviewer identify the strengths and weaknesses of the method/literature search?	39% 33–46	44% 37–50	0.38
4. Did the reviewer make useful comments on writing, organisation, tables and figures?	49% 42–56	55% 48–61	0.20
5. Were the reviewer’s comments constructive?	62% 55–68	65% 59–71	0.44
6. Did the reviewer supply appropriate evidence using examples from the MS to substantiate their comments?	51% 44–58	57% 50–63	0.18
7. Did the reviewer comment on the author’s interpretation of the results/literature?	39% 32–46	46% 39–52	0.15
8. How would you rate the tone of the review?	70% 64–76	69% 63–75	0.86
Overall proportions of all ratings 4 and 5	49% 46–51	55% 52–57	< 0.001

The quality of the reviews was assessed using the modified Review Quality Instrument [8]; scale 1–5, 5 excellent, 1 poor

**Table 3:** Quality assessment of the reviews conducted single-blind vs. double-blind; means, 95%CIs and SDs

	**Single-blind** (n 104)	**Double-blind** (n 116)	***P* value**
1. Did the reviewer discuss the importance of the research question/topic of the review?	3.38 3.22–3.54 SD 1.17	3.53 3.37–3.69 SD 1.22	0.19
2. Did the reviewer discuss the originality of the MS?	2.51 2.33–2.69 SD 1.34	2.90 2.72–3.07 SD 1.36	0.003
3. Did the reviewer identify the strengths and weaknesses of the method/literature search?	2.90 2.71–3.08 SD 1.33	3.13 2.97–3.30 SD 1.25	0.06
4. Did the reviewer make useful comments on writing, organisation, tables and figures?	3.31 3.16–3.46 SD 1.10	3.41 3.27–3.56 SD 1.10	0.31
5. Were the reviewer’s comments constructive?	3.61 3.50–3.73 SD 0.86	3.69 3.59–3.79 SD 0.80	0.35
6. Did the reviewer supply appropriate evidence using examples from the MS to substantiate their comments?	3.30 3.14–3.47 SD 1.22	3.43 3.28–3.58 SD 1.15	0.27
7. Did the reviewer comment on the author’s interpretation of the results/literature?	3.00 2.83–3.16 SD 1.22	3.21 3.06–3.35 SD 1.10	0.06
8. How would you rate the tone of the review?	3.79 3.70–3.89 SD 0.66	3.77 3.68–3.85 SD 0.65	0.69
Mean of assessments on all topics	3.22 3.17–3.28 SD 1.20	3.38 3.33–3.44 SD 1.13	< 0.001

The quality of the reviews was assessed using the modified Review Quality Instrument [8]; scale 1–5, 5 excellent, 1 poor

## Discussion

Switching to double-blind peer review did not alter the reviewers’ willingness to review. When reviewing double-blind, the reviewers’ recommendations were slightly more negative, and they more often suggested major revision and rejection. The overall quality of the double-blinded reviews, measured using the RQI modified to our purposes, was significantly better than that of single-blinded reviews.

The proportion of invitations that led to received review report when reviewing single-blinded was 67%, and when reviewing double-blinded, 66%. Other studies have observed much lower proportions of accepted review invitations. In a recent study, a general medical journal reported a proportion of successful review invitations to be 36% [11]. A study about six journals in ecology and evolution showed a decline from 56% of invitations generating a review in 2003 to 37% in 2015 [12]. Our reviewers’ higher willingness may be explained by their engagement to their “own” journal.

There are only few studies specifically exploring reviewers’ willingness to review in single- vs. double-blind setting. Huber et al. found that the reviewers significantly more often accepted the review invitation when the prominent researcher was shown as corresponding author [6]. On the other hand, when the review invitations with identified authors (either prominent or less-known researcher) were compared with anonymized invitations there was no difference in acceptance rate 32.5% vs 30.7%, respectively (*P* = 0.33). Another study in a setting when anonymization was voluntary found no evidence that the policy affected reviewer recruitment [13]. According to a questionnaire sent to reviewers in a Danish study, 38% preferred a double-blind review system, 34% preferred a single-blind system and 28% preferred an open review system [14]. In a British study, however, reviewers more often (35% vs 23%) declined to review when asked to be identified [8].

Interestingly, one study found that when reviewing double-blind, the reviewers less often recommended rejection [15]. In a study conducted in Ugeskrift for Læger, a journal published by the Danish Medical Association in Danish, anonymous reviewers more often recommended rejection [14]. This is in line with our findings, but unfortunately, our study was underpowered to show statistically significant results on this issue. The only significant difference was observed for recommending major revision. A recent meta-analysis [16], including 11 RCTs, found that the double-blind peer review process was associated with a lower rate of manuscript acceptance recommendations (14.2%) than the single-blind peer review process (19.0%).

Previous studies on double-blind peer review looking at the quality of peer review reports have given variable results. One study showed that blinding improved the quality of reviews [17] but, contrary to our findings, the majority of studies has not shown any improvement associated with double-blinding [18–20]. Moreover, a meta-analysis [21], including 3 randomised controlled trials (RCTs), evaluating the impact of double-blinding on the quality of the peer review reports found no effect. These studies examined only journals published in English. The Danish study on Ugeskrift for Læger showed no differences in review quality between double-blind reviews and reviews in which the reviewers’ identities were revealed [14].

### Limitations

The short study period which resulted to a relatively small material is a limitation of our study. It is uncertain if a larger data would have given different results, e.g., about the reviewers’ willingness to review. The time-window was limited because in March 2018 we changed our peer review platform, and the findings after that would not have been comparable to the earlier findings. On the other hand, we collected control data from two previous comparable periods, which data suggest that the changes in our peer review parameters were indeed associated with the switch. For the comparison, we chose the same months as the study months, in order to avoid seasonal variation, e.g., due to vacations of the reviewers, which might have affected the results.

Blinding probably failed in some cases, and the reviewers may have recognised the authors. Previous studies have shown that this may happen in up to 50% of cases [14–16], commonly due to self-citation and reviewer familiarity with authors’ work [22]. Unfortunately, we did not systematically collect data on how often our reviewers correctly guessed the identity of the authors. However, if some of the blinded reviewers had recognised the authors, this could only have diluted the findings, and the differences in the reviewer behaviour between single-blind and double-blind peer review may be even greater than those we observed.

Although being a validated tool [23], RQI also has limitations. While solely assessing the comments of the reviewer it cannot assess the accuracy of those comments in relation to the manuscript reviewed [24]. We modified the RQI to apply also to review manuscripts, but we did not test the reliability and validity of the modified RQI items for reviews, which is a limitation of our study. Another limitation is that we did not control for the quality of the manuscripts.

## Conclusions

We found that after switching from single-blind to double-blind peer review the quality of review reports, measured using the modified RQI, improved. The double-blinded reviewers more often discussed the originality of manuscripts, and the overall quality of reviews conducted double-blind was significantly better than those conducted single-blind. When performing a double-blind review, the reviewers’ recommendations were slightly more negative than those of single-blinded reviewers’, but the difference in the distribution of recommendations was not statistically significant. However, major revision was recommended more often during the double-blind period than during the control period (33% vs 23%). The reviewers’ willingness to review did not change.

We introduced double-blind peer review to the Finnish Medical Journal, in order to tackle the power imbalance between authors and reviewers, and the biases well-known in single-blind peer review. Our results indicate that double-blind peer review is a feasible model to a journal in a small language area without major downsides.

## Data Availability

Data are submitted to the Journal.

## Abbreviations

CI: Confidence interval
ICC: Intraclass correlation coefficient
IQR: Interquartile range
SD: Standard deviation
RCT: Randomized controlled trial
RQI: Review Quality Instrument
SD: Standard deviation
